# O17 - Assessment of efficacy and safety of sublingual tablets of house dust mite allergen extract in children and adolescents with allergic rhinitis

**DOI:** 10.1186/2045-7022-4-S1-O17

**Published:** 2014-03-14

**Authors:** Susanne Halken, Ulrich Wahn, Michel Mélac, Hélène Nguyen, Véronique Cadic, Robert K Zeldin

**Affiliations:** 1 Odense University Hospital, Boene Afd. H (Pediatric Dept H), Odense, Denmark; 2 Charite-Campus Virchow Klinikum, Klinik für Pädiatrie m. S. Pneumologie/Immunologie, Berlin, Germany; 3 Stallergenes S.A., Antony, France

## Background

The efficacy and safety of 500IR and 300IR sublingual tablets of house dust mite (HDM) allergen extracts have been demonstrated in adults with HDM-related allergic rhinitis (AR). Here, we report the efficacy and safety of the 300IR dose in a double-blind, placebo-controlled study in children and adolescents.

## Methods

Participants aged 5 to 17 years of age, with at least a 1-year history of HDM-associated AR requiring regular intake of symptomatic treatment, a positive skin prick test to HDM, HDM-specific serum IgE ≥0.7 kU/L, and an Average Rhinitis Total Symptom Score (ARTSS, scale: 0-12) ≥5 during a 7-day screening period were randomized (1:1) to receive 300IR or placebo tablets once daily for a year. Participants recorded their rhinitis symptoms and use of rescue medication. The primary efficacy endpoint, Average Adjusted Symptom Score (AAdSS), which adjusts symptom score for rescue medication use, was assessed over the last 2 months of the treatment period and analyzed by ANCOVA.

## Results

471 children and adolescents (300IR: 241, placebo: 230) were randomized. For the primary efficacy analysis, the AAdSS least-square mean difference between the 300IR and placebo groups was 0.01 (CI_95%_ [-0.41; 0.43]). Of note, the mean ARTSS in the placebo group was 6.7 at baseline, and dropped to 3.1 at Month 3, and to 2.3 at Month 12. The treatment was well tolerated. The most commonly reported adverse events (AEs) were application site reactions (oral pruritus, throat irritation), consistent with the safety profile observed in adults. No anaphylaxis or serious drug-related AEs were reported.

## Conclusions

In this study, participants were insufficiently symptomatic to enable evaluation of the efficacy of the HDM sublingual tablet. This suggests that, for clinical research purposes, inclusion of children with HDM-driven AR based on self-assessments of symptom severity and positive skin or in-vitro testing is inadequate. More stringent inclusion criteria must be considered in future studies.

